# The role of intestinal mucosa injury induced by intra-abdominal hypertension in the development of abdominal compartment syndrome and multiple organ dysfunction syndrome

**DOI:** 10.1186/cc13146

**Published:** 2013-12-09

**Authors:** Juntao Cheng, Zhiyi Wei, Xia Liu, Ximei Li, Zhiqiang Yuan, Jiang Zheng, Xiaodong Chen, Guangxia Xiao, Xiaoyi Li

**Affiliations:** 1 Department of Burn Intensive Care Unit, the 180th Hospital of Chinese People’s Liberation Army, Hua-yuan Road No.180, Quanzhou, Fujian Province 362000 P. R. China; 2 Department of Pharmacology, School of Pharmacy, the Second Military Medical University, Shanghai 200433, P. R. China; 3 Health Care Management Center, the 180th Hospital of Chinese People’s Liberation Army, Quanzhou, Fujian Province 362000 P. R. China; 4 State Key Laboratory of Trauma, Burns and Combined Surgery, Southwestern Hospital, the Third Military Medical University, Chongqing 400038, P. R. China; 5 Medical Research Center, Southwestern Hospital, the Third Military Medical University, Chongqing 400038, P. R. China; 6 Institute of Burn Research, Fuzhou Union Medical College Hospital, Fujian Medical University, Fuzhou, Fujian Province 350001, P. R. China

## Abstract

**Introduction:**

Abdominal distension is common in critical illness. There is a growing recognition that intra-abdominal hypertension (IAH) may complicate nonsurgical critical illness as well as after abdominal surgery. However, the pathophysiological basis of the injury to the intestinal mucosal barrier and its influence on the onset of abdominal compartment syndrome (ACS) and multiorgan dysfunction syndrome (MODS) remain unclear. We measured intestinal microcirculatory blood flow (MBF) during periods of raised intra-abdominal pressure (IAP) and examined how this influenced intestinal permeability, systemic endotoxin release, and histopathological changes.

**Methods:**

To test different grades of IAH to the injury of intestinal mucosa, 96 New Zealand white rabbits aged 5 to 6 months were exposed to increased IAP under nitrogen pneumoperitoneum of 15 mmHg or 25 mmHg for 2, 4 or 6 hours. MBF was measured using a laser Doppler probe placed against the jejunal mucosa through a small laparotomy. Fluorescein isothiocyanate (FITC)-conjugated dextran was administered by gavage. Intestinal injury and permeability were measured using assays for serum FITC-dextran and endotoxin, respectively, after each increase in IAP. Structural injury to the intestinal mucosa at different levels of IAH was confirmed by light and transmission electron microscopy.

**Results:**

MBF reduced from baseline by 40% when IAP was 15 mmHg for 2 hours. This doubled to 81% when IAP was 25 mmHg for 6 hours. Each indicator of intestinal injury increased significantly, proportionately with IAP elevation and exposure time. Baseline serum FITC-dextran was 9.30 (± SD 6.00) μg/ml, rising to 46.89 (±13.43) μg/ml after 15 mmHg IAP for 4 hours (*P* <0.01), and 284.59 (± 45.18) μg/ml after 25 mmHg IAP for 6 hours (*P* <0.01). Endotoxin levels showed the same pattern. After prolonged exposure to increased IAP, microscopy showed erosion and necrosis of jejunal villi, mitochondria swelling and discontinuous intracellular tight junctions.

**Conclusions:**

Intra-abdominal hypertension can significantly reduce MBF in the intestinal mucosa, increase intestinal permeability, result in endotoxemia, and lead to irreversible damage to the mitochondria and necrosis of the gut mucosa. The dysfunction of the intestinal mucosal barrier may be one of the important initial factors responsible for the onset of ACS and MODS.

## Introduction

Intra-abdominal hypertension (IAH) and abdominal compartment syndrome (ACS) are increasingly recognized as severe complications of critical illness [1-5]. Several studies have shown that IAH occurs not only in patients who have undergone abdominal surgery or sustained traumatic injuries [6,7], but can also complicate the course of nonsurgical critical illness such as severe burns and acute pancreatitis [8-10]. Thus, the secondary intra-abdominal hypertension is thought to be linked to both inflammation and fluid resuscitation [11].

Several prospective epidemiological studies of IAH and ACS in patients admitted to intensive care units (ICUs) have been conducted in different countries [12-14]. The World Society of Abdominal Compartment Syndrome (WSACS) has published a series of consensus statements on definitions, risk factors, measurement techniques, and recommendations for treatment and research [15,16], which were updated in 2013 [17]; however, given the high rate of morbidity and mortality, IAH and ACS still present particular challenges to clinicians. Previous studies have demonstrated that elevated intra-abdominal pressure (IAP) has deleterious effects on nearly all organ systems, including the cardiorespiratory, renal, gastrointestinal, neurologic, hepatic and adrenocortical systems [18]. The phenomenon of bacterial translocation (BT) as a consequence of gastrointestinal tract ischemia has also been described in animal models [19-22], but it is unclear whether IAH could provoke endotoxin release from gastrointestinal tract to blood circulation. Moreover, there is little research examining the influence of IAH in the genesis of intestinal mucosal barrier dysfunction, the role of intestinal mucosal injury in the development of ACS and multiple organ dysfunction syndrome (MODS), and furthermore the underlying histomorphological and ultrastructural features of mucosal injury are still not clear. Given the important role of endotoxin/bacterial translocation in the development of systematic inflammatory response syndrome (SIRS) and MODS [23], we hypothesized that a vicious cycle might exist between IAH and intestinal mucosal injury.

To this end, we examined the microcirculatory blood flow of the intestinal mucosa, intestinal permeability, endotoxin release into the systemic circulation and histopathological changes induced by IAH in a rabbit model. We measured these parameters at normal IAP, and at two elevated levels (15 mmHg and 25 mmHg), and discuss how these factors might influence the development of ACS and MODS.

## Materials and methods

### Animals

All experimental protocols were approved by the Animal Care and Use Committees of Fujian Medical University (Fuzhou, China) and of the 180th Hospital of the Chinese People’s Liberation Army (Quanzhou, China). Ninety-six healthy adult New Zealand white rabbits aged between 5 and 6 months, weighing 2.24 ± 0.19 kg (mean ± standard deviation, SD), (range 2.0 to 2.6 kg) were used in the study. All rabbits were housed in a caged facility under specific disease-free conditions.

### Rabbit model of intra-abdominal hypertension

The rabbit IAH model was established by means of a nitrogen gas pneumoperitoneum. Before surgery, rabbits were fasted overnight with free access to water. Anesthesia was induced using pentobarbital 30 mg/kg, administered via a marginal ear vein. Then, rabbits were placed supine on an operating table with a heating blanket to maintain core temperature between 36°C and 38°C. After the abdominal fur had been shaved and the skin sterilized with 75% medical grade ethanol, an intravenous catheter (18G, WEGO™, Weihai Wego Qiquan Medical Equipment Co., Ltd., Shandong, China) was used to penetrate the abdominal cavity in the caudal midline and sutured to the skin. An intra-abdominal pressure meter (0–5 kPa, Yea Thei Co., Ltd., Chia-I City, Taiwan) and a tube for delivering nitrogen gas were connected to the cannula via a three-way tap.

The rabbits were randomized into two groups: microcirculatory blood flow (MBF) assays were undertaken in 24; the other 72 were used to examine for intestinal injury and endotoxemia.

### Microcirculatory blood flow of intestinal mucosa

Twenty-four rabbits were further randomized into three subgroups: normal controls not exposed to elevated IAP (C), those exposed to an IAP of 15 mmHg (P15), and those exposed to an IAP of 25 mmHg (P25). Eight rabbits were allocated to each group, and MBF was measured at 2, 4 then 6 hours. To observe the microcirculatory blood flow in anesthetized rabbits, a 3 to 4 cm laparotomy incision was made in the midline of the upper abdomen. A laser Doppler probe (PeriFlux5000™, Perimed AB, Järfälla, Sweden) was inserted through a small incision in the jejunum 5 cm below the ligament of Treitz and sutured to the jejunal mucosa. We ensured that the tip of the probe remained continuously and steadily in contact with the mucosa to prevent motion artifact with peristalsis or changes in IAP. After the abdomen was closed with sutures, nitrogen gas was insufflated to increase the IAP to the target level of each group over approximately 10 minutes; that pressure was maintained throughout the remainder of the experiment. MBF measurements from the probe were displayed on a monitor and recorded on a computer. At the end of the allotted time periods for each group data were recorded and expressed in perfusion units (PU). In the control groups, all procedures were performed apart from the insufflation of nitrogen.

### Evaluation of intestinal mucosal injury induced by IAH

Seventy-two rabbits were further randomized into one of nine groups so that there were eight rabbits in each: groups were exposed to IAPs of nil (baseline control, C), 15 mmHg and 25 mmHg for periods of 2, 4 or 6 hours; groups were identified as C-2, 4, 6; P15-2, 4, 6; P25-2, 4, 6 respectively. Injury to the intestinal mucosal barrier was assessed by means of measuring its permeability to fluorescein isothiocyanate-conjugated dextran (FITC-dextran, 4 kDa), the concentration of endotoxin in plasma, and histological findings.

FITC-dextran (4 kDa, Sigma-Aldrich, St Louis, MO, USA) was diluted with sterile 0.9% NaCl solution to a final concentration of 100 mg/ml prior to injection and kept in the dark. Beforehand a gastric and duodenal tube (F6, HOSHIN™, Nanjing Hoshin Medical Instrument Co., Ltd., Nanjing, China) had been inserted via the rabbit’s mouth. Thirty minutes before the IAP period had elapsed; 250 mg/kg body weight of the FITC-dextran solution was injected into the duodenal lumen. At the end of the period of increased IAP, nitrogen gas was removed and a laparotomy was performed. Two samples of blood were collected from the hepatic portal vein, one for an endotoxin assay, and the other for a FITC-dextran assay. A 1 to 2 cm segment of jejunum was excised for histological examination.

#### Measurement of intestinal mucosa-to-blood permeability

One hepatic portal venous blood sample was placed into heparinized tubes and centrifuged at 10,000 *g* for 10 min. Plasma was removed and subsequently assayed using a fluorescence spectrophotometer (F-2500, Hitachi, Tokyo, Japan) with an excitation wavelength of 480 nm and an emission wavelength of 520 nm. To determine the concentration of FITC-dextran, a standard curve was obtained through serial dilution in normal rabbit plasma.

#### Assay for endotoxin in blood plasma

Plasma lipopolysaccharide (LPS) levels were quantified using Limulus amebocyte lysate (LAL) assay kits (Gulangyu™, Chinese Horseshoe Crab Reagent Manufactory Co., Ltd., Xiamen, China) on an absorbance microplate reader (Elx808IU, BioTek, Winooski, VT USA) according to the manufacturer’s instructions. All consumables including micropipette tips, tubes and water were certified sterile and pyrogen-free to avoid contamination. Briefly, the blood sample was added to pyrogen-free tubes and centrifuged; plasma was collected and aliquots were stored at −70°C until assay. A standard curve was made using the standardized LPS provided in the kits and concentrations calculated according to the LAL assay protocol.

#### Pathological changes observed by light and transmission electron microscopy

Intestinal segments were washed three times using PBS at 4°C, and then divided into two parts for examination by light and transmission electron microscopy (TEM). Optical microscopy samples were fixed in 10% formalin, dehydrated through a graded series of ethanol, embedded in paraffin and cut into 3 to 5 μm sections. These were stained using hematoxylin and eosin (H&E) and viewed (CX21BIM-SET5, Olympus Corp., Tokyo, Japan). Samples for TEM were fixed with glutaraldehyde and osmium tetroxide, embedded in epoxy resin and cut into ultra-thin sections before examination (Jem-2100, Jeol Ltd., Tokyo, Japan).

### Statistics

Statistical analysis was performed with IBM SPSS Statistics (version 20.0, IBM Corp., Armonk, NY, USA). The one-way ANOVA and Bonferroni or Tamhane’s T2 methods were used to compare variance between groups. Data are presented as mean ± standard deviation (SD), *P* <0.05 was considered significant.

In a one-way ANOVA study, sample sizes of eight per group were obtained for the three groups whose means were to be compared. The total sample of 24 subjects achieves 83% power to detect differences among the means using an F test with a 0.05 significance level. The size of the variation in the means is represented by their SD (8.16). The common standard deviation within a group is assumed to be 11.50.

## Results

### Influence of IAH on microcirculatory blood flow in intestinal mucosa

**Figure 1:**
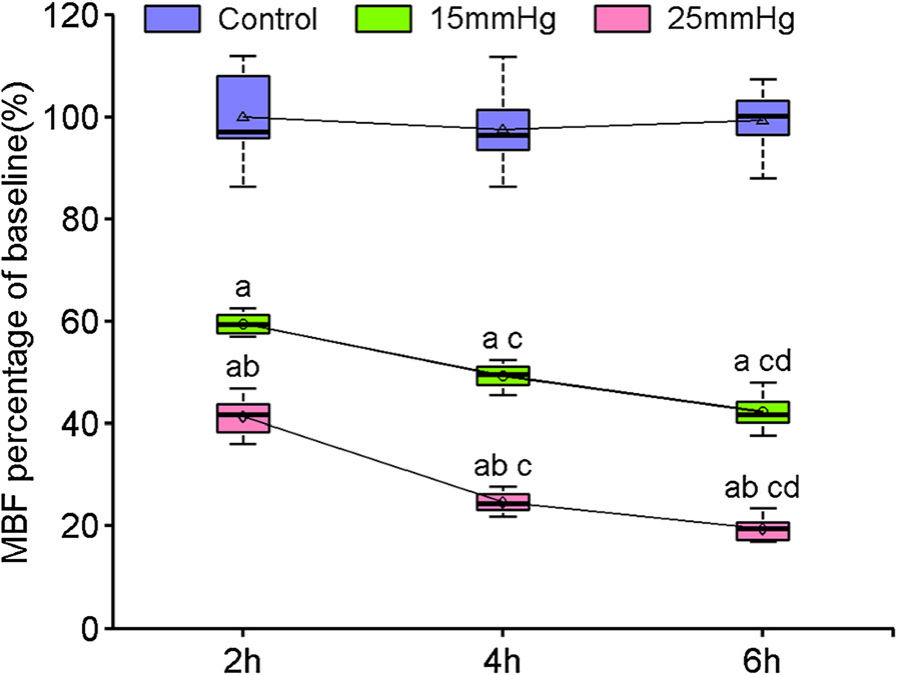
**The influence of two levels of intra-abdominal pressure (15 and 25 mmHg) on jejunal mucosal microcirculatory blood flow (MBF), after 2, 4 and 6 hours.** Data are presented as mean ± SD (n = 8) and compared by one-way ANOVA and Bonferroni or Tamhane’s T2 methods: ^a^*P* <0.01 versus control; ^b^*P* <0.01 versus 15 mmHg; ^c^*P* <0.01 versus 2 hrs; ^d^*P* <0.01 versus 4 hrs. No significant differences were found between control groups (*P* = 0.80).

The MBF measured by laser Doppler flowmetry are expressed as a percentage of baseline flow and are shown in Figure 1 and Table S1 in Additional file 1. Substantial reductions in MBF could be seen at the lower IAP even at 2 hours, reductions that became more profound during longer exposures. MBF decreased from baseline by approximately 40%, 50% and 58% in P15-2, 4, 6 groups respectively compared with control (*P* <0.01). Further reductions were seen in the P25-2, 4, 6 groups: MBF decreased by approximately 60%, 76% and 81% from baseline (*P* <0.01).

### Increased intestinal mucosa-to-blood permeability

**Figure 2:**
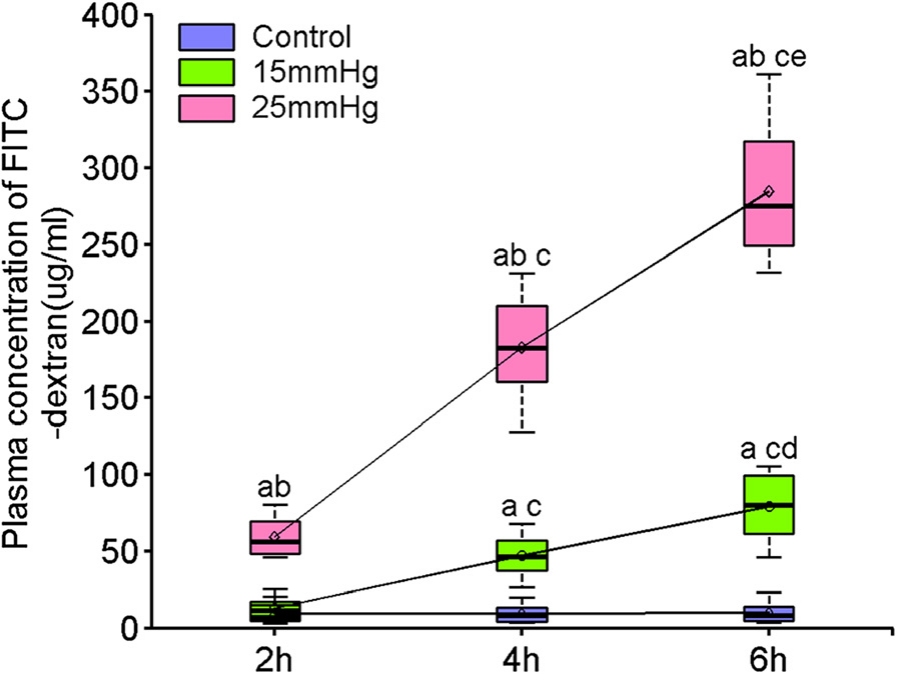
**Alterations in intestinal permeability induced by two levels of intra-abdominal pressure (15 and 25 mmHg) after 2, 4 and 6 hours of exposure.** Data are presented as mean ± SD (n = 8) and compared by one-way ANOVA and Bonferroni or Tamhane’s T2 methods: ^a^*P* <0.01 versus control; ^b^*P* <0.01 versus 15 mmHg; ^c^*P* <0.01 versus 2 hrs; ^d^*P* <0.05 versus 4 hrs; ^e^*P* <0.01 versus 4 hrs. No significant differences were seen between control groups (*P* = 0.98).

FITC-dextran levels (shown in Figure 2 and Table S2 in Additional file 2) were lowest in the control group at 2, 4 and 6 hours with no significant difference between each time point (*P* = 0.98). The concentration of FITC-dextran in the P15-2 group showed no significant difference compared with normal controls (*P* = 0.72), but increased significantly in the subsequent P15-4 and P15-6 groups (*P* <0.01). FITC-dextran levels increased significantly in all three P25 groups. At the end of 6 hours’ exposure to an IAP of 25 mmHg, FITC-dextran levels increased 28-fold above those of the control group.

### Endotoxin transmitted from the intestinal mucosa to blood

**Figure 3:**
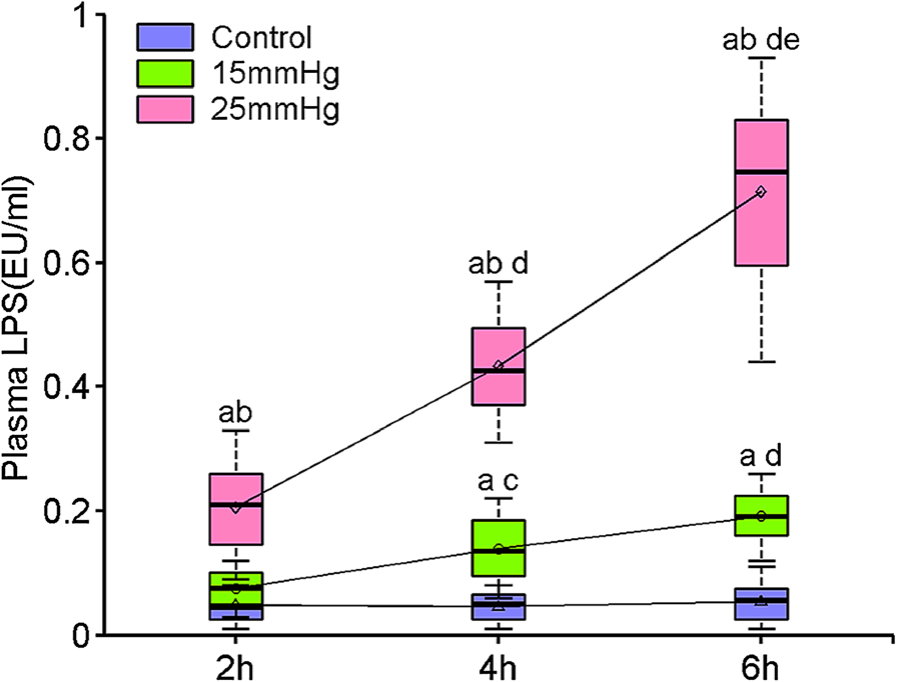
**Alterations in blood endotoxin induced by two levels of intra-abdominal pressure (15 and 25 mmHg), after 2, 4 and 6 hours of exposure.** Data are presented as mean ± SD (n = 8) and compared by one-way ANOVA and Bonferroni or Tamhane’s T2 methods: ^a^*P* <0.01 versus control; ^b^*P* <0.01 versus 15 mmHg; ^c^*P* <0.01 versus 2 hrs; ^d^*P* <0.01 versus 2 hrs; ^e^*P* <0.01 versus 4 hrs. No significant differences were seen between control groups (*P* = 0.86).

LPS levels in the normal control group were very low and showed no significant differences between each time point (*P* = 0.86). The concentration of LPS in the P15-2 group showed no significant difference to normal controls (*P* = 0.23), but increased significantly in the subsequent P15-4 and P15-6 groups (*P* <0.01), which reflected a trend very similar to that of the changes in FITC-dextran concentration. LPS level increased significantly in all three P25 groups; there was a more than three-fold increase over the P15 groups (*P* <0.01). At the end of 6 hours of exposure to an IAP of 25 mmHg, LPS levels were more than 13 times that of controls (Shown in Figure 3 and Table S3 in Additional file 3).

### Pathological changes observed by light and transmission electron microscopy

**Figure 4:**
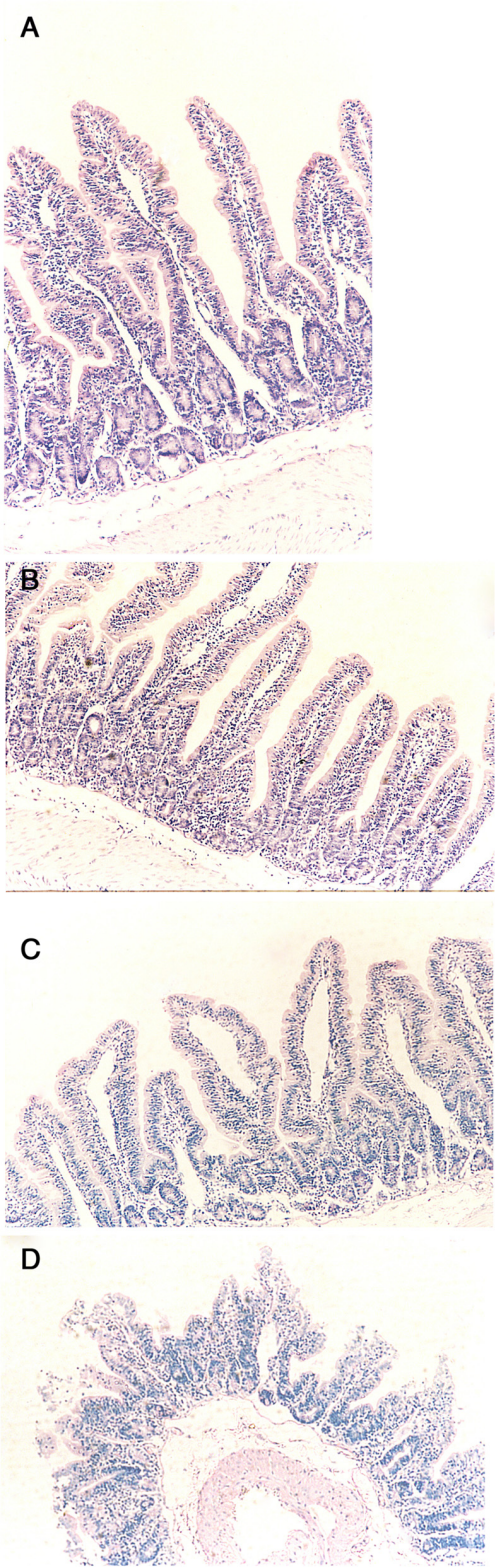
**Pathological changes of intestinal mucosa observed by light microscopy. (A)** Normal control; **(B)** after an intra-abdominal pressure (IAP) of 15 mmHg for 4 hrs, jejunal villi were moderately edematous, mild neutrophil infiltration can be seen, but gross integrity was relatively maintained; **(C)** after an IAP of 25 mmHg for 2 hrs, obvious neutrophil infiltration and central lacteal expansion were seen, along with thickening of the walls of the villi; **(D)** after an IAP of 25 mmHg for 4 hrs, extensive jejunal villi erosion and necrosis were seen. (Magnification for each: ×100).

In the group exposed to an IAP of 15 mmHg, jejunal villi were moderately edematous with mild infiltration of neutrophils, but gross integrity was relatively preserved (Figure 4B). Infiltration of neutrophils, central lacteal expansion and thickening of the walls of the villi were more obvious in rabbits exposed to an IAP of 25 mmHg for 2 hours (Figure 4C) than specimens taken from controls (Figure 4A) or those exposed to the lower pressure. Extensive erosion and necrosis of the jejunal villi were seen in the group exposed to an IAP of 25 mmHg for more than 4 hours (Figure 4D).

**Figure 5:**
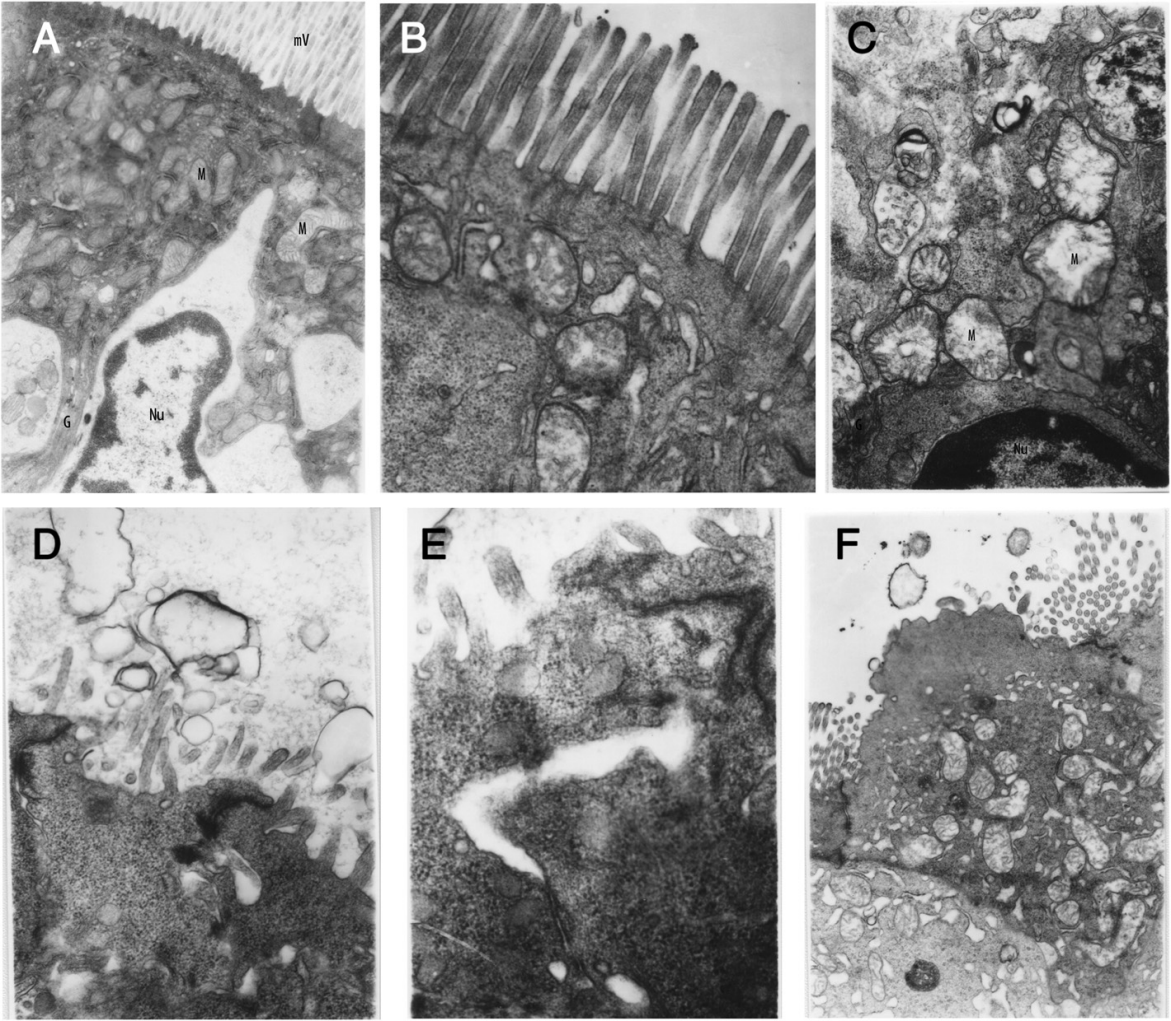
**Pathological changes of intestinal mucosa observed by transmission electron microscopy. (A)** Mitochondria (M) and microvilli (mV) in a normal jejunal mucosal epithelial cell (×10,000); **(B)** mildly swollen mitochondria but intact microvilli can be seen after an intra-abdominal pressure (IAP) of 15 mmHg for 4 hrs (×14,000); **(C and D)** severe mitochondrial swelling (M) and disruption of the cristae, and shortened, irregular and vacuolated microvilli can be seen after an IAP of 25 mmHg for 2 hrs (**C**: ×14,000, **D**: ×17,000); **(E and F)** discontinuous cell tight junctions, mitochondrial ballooning, degeneration and necrosis, and vacuolated and necrotic microvilli were seen that had begun to flake from the surface of membrane after an IAP of 25 mmHg for 6 hrs (**E**: ×20,000, **F**: ×7,000).

On TEM, mild mitochondrial swelling could be seen in the IAP 15 mmHg for the 4-hour group (Figure 5B), and microvilli remained intact when compared with normal microstructures (Figure 5A). In the group exposed to an IAP of 25 mmHg for 2 hours, more extensive mitochondrial swelling could be seen with disruption of the cristae (Figure 5C), and shortening and irregularity of the microvilli (Figure 5D). In the group exposed to an IAP 25 mmHg for 6 hours, cell-cell tight junctions were discontinuous (Figure 5E), cristae had separated from the mitochondrial membrane and dissolved, mitochondrial degeneration and engulfment could be seen, and microvilli were vacuolated and had flaked from the surface of membrane (Figure 5F).

## Discussion

The gut has long being regarded as a ‘trigger’ or ‘promoter’ of sepsis and multiorgan failure (MOF); the important role of gut dysfunction has been illustrated in numerous studies [24-27]. Marshall aptly described the gastrointestinal tract as an ‘undrained abscess’ [28]. IAH could be either a cause or a consequence of gastrointestinal mucosal injury, with the potential for a vicious cycle to develop in critically ill patients when IAP rises and causes ACS, which in turn worsens mucosal injury. We studied the influence of raised IAP on MBF and sought clues as to the pathophysiological mechanisms that underpin bacterial translocation.

Animal models are often used to study IAH and ACS. IAP has been manipulated by means of air, carbon dioxide and nitrogen pneumoperitoneum, instillation of fluids, and insertion of balloons or solid materials [29-33]. Although none can completely reproduce the real-life pathophysiological processes triggered by capillary leak and fluid accumulation, they can at least shed light upon the influence of IAP on end organ function. We used nitrogen gas as it is inert, harmless and inexpensive, is minimally absorbed allowing IAP to be maintained easily, does not result in acidosis or excessive fluid absorption by the peritoneum, and is distributed evenly throughout the peritoneum.

Previous studies have shown that IAH results in a significant decrease in cardiac output, mean blood pressure, and blood flow in the superior mesenteric arterial and intestinal mucosa [34-37]. Our data confirm that blood flow is also diminished in the intestinal mucosal microcirculation, by approximately half when IAP is 15 mmHg and substantially more when IAP is 25 mmHg, with further reductions seen when exposure to raised IAP is longer. The extent of this reduction creates local ischemia and hypoxia, which, when persistent, causes further mucosal injury.

As well as impeding MBF, IAH causes a distinct increase in intestinal mucosal permeability. It has been shown that abnormal and severe increases in the intestinal permeability are associated with the onset of MODS in critically ill patients [38]. Intestinal permeability refers to the ability of large molecules (>150 Da) to cross the mucosa. A 4 kDa FITC-dextran was used in this experiment to examine the permeability of jejunal mucosa provoked by IAH. We found significant elevation of plasma FITC-dextran with increasing IAP and duration of exposure, to the extent that a 28-fold increase was seen in the group exposed to the highest IAP for the longest time compared with controls. Increased permeability, and destruction of the mechanical and immunological barrier that follows mucosal injury, provides the opportunity for BT and endotoxin release into the systemic circulation. It is widely recognized that LPS activates vascular endothelial cells, mononuclear macrophages, and lymphocytes among others, resulting in the release of a cascade of cytokines and other proinflammatory or inflammatory substances, which themselves play a vital role in the genesis and development of systemic inflammatory response syndrome, sepsis and organ dysfunction or failure [39-41]. These cytokines and neutrophil migration conversely stimulate vascular endothelial cells, leading to ‘capillary leak syndrome’. As this pathophysiologic process has some analogy with acute lung injury (ALI) or acute renal injury (ARI), it has been suggested that the terms acute intestinal distress syndrome (AIDS) or acute intestinal permeability syndrome (AIPS) are used to describe the intestinal dysfunction resulting from shock or ischemia [42-44].

IAH and ACS were associated with significantly higher endotoxin exposure in critically ill patients with severe acute pancreatitis or abdominal sepsis and suggestive of gut barrier dysfunction [26]. Substantial BT across the intestinal wall and mesenteric lymph nodes was observed when IAP increased to 15 and 30 mmHg in a porcine model of IAH [22]; however, elevated plasma levels of endotoxin were not detected. We found unequivocal elevation of LPS levels in blood sampled from the portal vein, and the levels correlated with IAP and duration of exposure. This might suggest a possible link between IAH-induced intestinal mucosal injury and MODS.

Light and electron microscopy further confirmed the nature of the injury to the intestinal mucosa. At lower IAP, the villi were moderately edematous and there was mild infiltration of neutrophils, but gross integrity was preserved. Extensive erosion and necrosis of the villi were seen at higher IAP. At the ultrastructural level, lower IAP resulted in swelling of the mitochondria in jejunal mucosal epithelial cells, while the microvilli were essentially structurally normal. With longer exposure and higher pressure, extensive swelling, vacuolated mitochondria and shortened irregular microvilli could be seen. In the group exposed to the highest IAP for longest, the cristae had separated from the mitochondrial membrane and dissolved, mitochondria had degenerated and been engulfed, microvilli had flaked from the cell membrane and the tight junctions had separated.

Taken as a whole, these results suggest that IAH can significantly decrease the MBF of gut mucosa and cause mucosal edema. These factors may accelerate ischemic damage to the gut when IAH becomes persistent. Without an effective means of intervening, and after the IAP has been 25 mmHg for more than 4 hours, mucosal villi become necrotic and the onset of BT and endotoxin release to the systemic circulation would greatly increase the possibility of the occurrence of sepsis and MODS, which will further aggravate and complicate the underlying disease process. Therefore, to ameliorate damage to the gut mucosa, and to break the vicious cycle that leads to sepsis and MODS, maintaining the function of the intestinal mucosal barrier is essential, even in the early stages of IAH.

## Conclusions

Intestinal mucosal MBF and permeability can be significantly affected by IAPs of 15 mmHg and 25 mmHg. Furthermore, IAH can cause endotoxin release into the systemic circulation, and the level of endotoxin increases with IAP and its duration. Histological and ultrastructural examination further confirms the nature of the intestinal mucosal injury induced by IAH, and seems to increase in a dose-dependent fashion in terms of IAP level and duration. The dysfunction of the intestinal mucosal barrier may be one of the important initial factors responsible for the onset of ACS and MODS, and suggests that the protection of intestinal mucosa may be a valuable target against intra-abdominal hypertension in critical illness.

## Key messages

•IAH can substantially reduce intestinal mucosal microcirculatory blood flow - by as much as 80% when rabbits are exposed to an IAP of 25 mmHg for 6 hours.

•Intestinal mucosal permeability increases with elevated IAP and prolonged exposure.

•IAH results in endotoxin release to the systemic circulation; the concentration of plasma endotoxin correlates with IAP and duration of exposure.

•The intestinal mucosal injury induced by IAH is evident on histological and ultrastructural examination.

## Abbreviations

ACS: Abdominal compartment syndrome; BT: Bacterial translocation; FITC-dextran: Fluorescein isothiocyanate-conjugated dextran; H&E: Hematoxylin and eosin; IAH: Intra-abdominal hypertension; IAP: Intra-abdominal pressure; LAL: Limulus amebocyte lysate; LPS: Lipopolysaccharide; MBF: Microcirculatory blood flow; MODS: Multiple organ dysfunction syndrome; TEM: Transmission electron microscopy; WSACS: World Society of Abdominal Compartment Syndrome.

## Competing interests

The authors declare that they have no competing interests.

## Authors’ contributions

JC conceived and designed the study. ZW and XiaL performed the measurement of intestinal mucosa permeability. XimL, ZY and JZ performed the assay of endotoxin in blood plasma. JC, XC and XiaoL observed the histopathological changes by light and transmission electron microscopy. ZY and GX participated in the design of the study and performed the statistical analysis. JC wrote the manuscript and it was revised by GX. ZW, XiaL and XimL helped to draft the manuscript. All authors have read and approved the manuscript for publication.

## Supplementary Material

Additional file 1: Table S1 The influence of two levels of intra-abdominal pressure (15 and 25 mmHg) on jejunal mucosal microcirculatory blood flow (MBF), after 2, 4 and 6 hours. Data are presented as mean ± SD (n = 8) and compared by one-way ANOVA and Bonferroni or Tamhane’s T2 methods: ^a^*P* <0.01 versus control; ^b^*P* <0.01 versus 15 mmHg; ^c^*P* <0.01 versus 2 hrs; ^d^*P* <0.01 versus 4 hrs. No significant differences were found between control groups (*P* = 0.80). Click here for file

Additional file 2: Table S2 Alterations in intestinal permeability induced by two levels of intra-abdominal pressure (15 and 25 mmHg) after 2, 4 and 6 hours of exposure. Data are presented as mean ± SD (n = 8) and compared by one-way ANOVA and Bonferroni or Tamhane’s T2 methods: ^a^*P* <0.01 versus control; ^b^*P* <0.01 versus 15 mmHg; ^c^*P* <0.01 versus 2 hrs; ^d^*P* <0.05 versus 4 hrs; ^e^*P* <0.01 versus 4 hrs. No significant differences were seen between control groups (*P* = 0.98). Click here for file

Additional file 3: Table S3 Alterations in blood endotoxin induced by two levels of intra-abdominal pressure (15 and 25 mmHg), after 2, 4 and 6 hours of exposure. Data are presented as mean ± SD (n = 8) and compared by one-way ANOVA and Bonferroni or Tamhane’s T2 methods: ^a^*P* <0.01 versus control; ^b^*P* <0.01 versus 15 mmHg; ^c^*P* <0.01 versus 2 hrs; ^d^*P* <0.01 versus 2 hrs; ^e^*P* <0.01 versus 4 hrs. No significant differences were seen between control groups (*P* = 0.86). Click here for file
