# Supplementary material for: The role of intestinal mucosa injury induced by intra-abdominal hypertension in the development of abdominal compartment syndrome and multiple organ dysfunction syndrome
Source: Crit Care. 2013 Dec 9;17(6):R283. doi: 10.1186/cc13146 (PMC4057115; doi:10.1186/cc13146)
Supplement: Additional file 1: Table S1 — The influence of two levels of intra-abdominal pressure (15 and 25 mmHg) on jejunal mucosal microcirculatory blood flow (MBF), after 2, 4 and 6 hours. Data are presented as mean ± SD (n = 8) and compared by one-way ANOVA and Bonferroni or Tamhane’s T2 methods: aP <0.01 versus control; bP <0.01 versus 15 mmHg; cP <0.01 versus 2 hrs; dP <0.01 versus 4 hrs. No significant differences were found between control groups (P = 0.80). [file cc13146-S1.doc]

|  | 2hrs | 4hrs | 6hrs |
| --- | --- | --- | --- |
| C (%) | 100.01±8.52 | 97.57±7.70 | 99.37±5.94 |
| P15 (%) | 59.55±4.07^a^ | 49.35±4.37^ac^ | 42.27±5.25^acd^ |
| P25 (%) | 41.34±5.61^ab^ | 24.65±4.01^abc^ | 19.42±4.29^abcd^ |

**Table 1** The influence of two levels of intra-abdominal pressure (15 and 25 mmHg) on jejunal mucosal microcirculatory blood flow (MBF), after 2, 4 and 6 hours. Data are presented as mean ± SD (n = 8) and compared by one-way ANOVA and Bonferroni or Tamhane’s T2 methods: ^a^ p < .01 versus control; ^b^ p < .01 versus 15 mmHg; ^c^ p < .01 versus 2 hrs; ^d^ p < .01 versus 4 hrs. No significant differences were found between control groups (p = .80).
