# Supplementary material for: The role of intestinal mucosa injury induced by intra-abdominal hypertension in the development of abdominal compartment syndrome and multiple organ dysfunction syndrome
Source: Crit Care. 2013 Dec 9;17(6):R283. doi: 10.1186/cc13146 (PMC4057115; doi:10.1186/cc13146)
Supplement: Additional file 2: Table S2 — Alterations in intestinal permeability induced by two levels of intra-abdominal pressure (15 and 25 mmHg) after 2, 4 and 6 hours of exposure. Data are presented as mean ± SD (n = 8) and compared by one-way ANOVA and Bonferroni or Tamhane’s T2 methods: aP <0.01 versus control; bP <0.01 versus 15 mmHg; cP <0.01 versus 2 hrs; dP <0.05 versus 4 hrs; eP <0.01 versus 4 hrs. No significant differences were seen between control groups (P = 0.98). [file cc13146-S2.doc]

|  | 2 hrs | 4 hrs | 6 hrs |
| --- | --- | --- | --- |
| C (ug/ml) | 9.51 ± 6.69 | 9.30 ± 6.00 | 10.00 ± 6.69 |
| P15 (ug/ml) | 12.83 ± 6.88 | 46.89 ± 13.43^ac^ | 79.10 ± 21.76^acd^ |
| P25 (ug/ml) | 59.19 ± 12.53^ab^ | 183.10 ± 33.76^abc^ | 284.59 ± 45.18^abce^ |

**Table 2** Alterations in intestinal permeability induced by two levels of intra-abdominal pressure (15 and 25 mmHg) after 2, 4 and 6 hrs of exposure. Data are presented as mean ± SD (n = 8) and compared by one-way ANOVA and Bonferroni or Tamhane’s T2 methods: ^a^ p < .01 versus control; ^b^ p < .01 versus 15 mmHg; ^c^ p < .01 versus 2 hrs; ^d^ p < .05 versus 4 hrs; ^e^ p < .01 versus 4 hrs. No significant differences were seen between control groups (p = 0.98).
