# Supplementary material for: The role of intestinal mucosa injury induced by intra-abdominal hypertension in the development of abdominal compartment syndrome and multiple organ dysfunction syndrome
Source: Crit Care. 2013 Dec 9;17(6):R283. doi: 10.1186/cc13146 (PMC4057115; doi:10.1186/cc13146)
Supplement: Additional file 3: Table S3 — Alterations in blood endotoxin induced by two levels of intra-abdominal pressure (15 and 25 mmHg), after 2, 4 and 6 hours of exposure. Data are presented as mean ± SD (n = 8) and compared by one-way ANOVA and Bonferroni or Tamhane’s T2 methods: aP <0.01 versus control; bP <0.01 versus 15 mmHg; cP <0.01 versus 2 hrs; dP <0.01 versus 2 hrs; eP <0.01 versus 4 hrs. No significant differences were seen between control groups (P = 0.86). [file cc13146-S3.doc]

|  | 2hrs | 4hrs | 6hrs |
| --- | --- | --- | --- |
| C (EU/ml) | 0.048±0.027 | 0.046±0.025 | 0.054±0.033 |
| P15 (EU/ml) | 0.075±0.032 | 0.139±0.056^ac^ | 0.191±0.046^ad^ |
| P25 (EU/ml) | 0.206±0.081^ab^ | 0.433±0.086^abd^ | 0.714±0.167^abde^ |

**Table 3** Alterations in blood endotoxin induced by two levels of intra-abdominal pressure (15 and 25 mmHg), after 2, 4 and 6 hrs of exposure. Data are presented as mean ± SD (n = 8) and compared by one-way ANOVA and Bonferroni or Tamhane’s T2 methods: ^a^ p < .01 versus control; ^b^ p < .01 versus 15 mmHg; ^c^ p < .01 versus 2 hrs; ^d^ p < .01 versus 2 hrs; ^e^ p < .01 versus 4 hrs. No significant differences were seen between control groups (p = 0.86).
